# Prognostic Significance of TBX21 and GATA3 Subtype Classification in Indolent Adult T‐Cell Leukemia‐Lymphoma With Cutaneous Lesions

**DOI:** 10.1111/1346-8138.17917

**Published:** 2025-08-20

**Authors:** Kazuhiro Kawai, Youhei Uchida, Takuro Kanekura

**Affiliations:** ^1^ Department of Dermatology Kagoshima University Graduate School of Medical and Dental Sciences Kagoshima Japan; ^2^ Department of Dermatology Kido Hospital Niigata Japan

**Keywords:** adult T‐cell leukemia/lymphoma, ATLL, immunophenotyping, prognosis

## Abstract

Chronic‐type adult T‐cell leukemia‐lymphoma (ATL) without unfavorable prognostic factors and smoldering‐type ATL are classified as indolent ATL. Indolent ATL patients often present with cutaneous lesions, but their clinical course is diverse. Smoldering‐type ATL presenting with tumoral cutaneous lesions and without lung involvement has a poor prognosis and is designated as primary cutaneous tumoral (PCT) type. Indolent ATL prognostic index (iATL‐PI) based on the soluble IL‐2 receptor levels and its modified version have been proposed. Peripheral T‐cell lymphoma, not otherwise specified (PTCL NOS) is classified into two prognostic subtypes, PTCL‐TBX21 and PTCL‐GATA3, using an immunohistochemistry (IHC) algorithm based on TBX21/CXCR3 and GATA3/CCR4 expression. Although ATL can also be classified into TBX21 and GATA3 subtypes, the prognostic significance of this classification in ATL remains unclear. To evaluate the prognostic significance of the IHC‐based classification of TBX21 and GATA3 subtypes in indolent ATL with cutaneous lesions, we performed a single‐center, retrospective cohort study involving 47 patients with indolent ATL with cutaneous lesions. We estimated survival according to the patients' characteristics, including the IHC subtype, and assessed their prognostic significance. Patients with GATA3 subtype showed significantly worse survival than those with TBX21 subtype. In multivariate analyses, GATA3 subtype was identified as an independent prognostic factor for overall survival, along with older age, PCT type, and modified iATL‐PI. GATA3 subtype was also independently associated with poorer progression‐free survival, in addition to PCT type and modified iATL‐PI. These results indicate that the IHC‐based classification into TBX21 and GATA3 subtypes is useful for predicting survival and progression in indolent ATL patients with cutaneous lesions.

## 1 Introduction

Adult T‐cell leukemia‐lymphoma (ATL) is a mature T‐cell neoplasm associated with human T‐lymphotropic (or T‐cell leukemia) virus type I (HTLV‐1) [1]. ATL has been classified into four clinical subtypes: acute, lymphoma, chronic, and smoldering [2]. The chronic type is further divided into unfavorable and favorable subtypes based on the presence or absence of either of three unfavorable prognostic factors: serum blood urea nitrogen or lactate dehydrogenase levels higher than the normal upper limit or albumin levels lower than the normal lower limit [3]. Acute, lymphoma, and unfavorable chronic types are categorized as aggressive ATL, whereas favorable chronic and smoldering types are classified as indolent ATL.

Aggressive ATL is treated with intensive chemotherapy or chemoimmunotherapy, followed by allogeneic hematopoietic stem cell transplantation in eligible patients [4, 5]. Interferon‐α with zidovudine (IFN/AZT) has shown activity in indolent ATL, but its role in aggressive ATL remains uncertain [6, 7, 8], and it is not currently approved in Japan.

Indolent ATL generally progresses slowly, but the long‐term prognosis of indolent ATL patients remains poor [9, 10]. Nearly half of patients progress to aggressive ATL within five years [1]. Chemotherapy is generally withheld until progression to aggressive ATL, except in patients with progressive disease [4, 5]. However, the clinical presentation and course of indolent ATL are highly variable [9]. Where IFN/AZT is unavailable, recommended treatment strategies for favorable chronic‐type and symptomatic smoldering‐type (e.g., with cutaneous lesions) vary from skin‐directed therapies to chemotherapy, depending on clinical presentation and course [4, 11, 12].

Soluble IL‐2 receptor (sIL‐2R) was identified as a sole independent prognostic factor for chronic and smoldering types of ATL in a nationwide survey conducted in Japan [13]. To enable risk‐adapted treatment strategies, the indolent ATL prognostic index (iATL‐PI) based on the sIL‐2R levels was developed [13]. Using cutoff values of 1000 and 6000 U/mL, patients are classified into low‐, intermediate‐, and high‐risk groups. Although the usefulness of iATL‐PI has been validated in subsequent studies [14, 15], more than half of patients are classified into the intermediate‐risk group [13, 14, 15]. To improve risk stratification, modified iATL‐PI has been proposed, which subdivides the intermediate‐risk group of the original iATL‐PI into low‐intermediate‐ and high‐intermediate‐risk groups using an additional cutoff value of 3000 U/mL [16].

Approximately half of ATL patients present with cutaneous lesions [1]. Conflicting results have been reported on the prognostic significance of the presence of cutaneous lesions [10, 17, 18, 19, 20, 21]. Controversy also exists over the types of cutaneous lesions associated with worse prognosis [9, 19, 20, 22, 23, 24]. However, ATL patients with tumoral cutaneous lesions and without systemic involvement frequently show a rapid clinical course and have a poor prognosis [15, 18, 20, 21]. These cases have been proposed to be classified as a distinct clinical subtype termed primary cutaneous tumoral (PCT) type, which is defined as the presence of cutaneous tumors in the absence of lymphocytosis, hypercalcemia, and involvement of lymph nodes or internal organs (i.e., smoldering‐type ATL with tumoral cutaneous lesions and without lung involvement) [18, 21]. Smoldering‐type ATL with cutaneous tumors may also be regarded as lymphoma‐type ATL, extranodal primary cutaneous variant, if histopathological findings are high‐grade T‐cell lymphoma and there are no abnormal lymphocytes in the peripheral blood (≤ 1%) [25]. It is recommended that PCT‐type ATL be treated as aggressive ATL [4]. However, PCT‐type ATL does not always follow an aggressive clinical course or require intensive chemotherapy.

Gene expression profiling has identified two molecular subtypes of peripheral T‐cell lymphoma, not otherwise specified (PTCL NOS): PTCL‐TBX21 and PTCL‐GATA3, characterized by overexpression of the Th1‐ and Th2‐transcription factors TBX21 and GATA3, respectively, and corresponding target genes [26]. They are dependent on distinct oncogenic pathways and have different prognoses. These two prognostic subtypes can be classified using an immunohistochemistry (IHC) algorithm based on TBX21/CXCR3 and GATA3/CCR4 expression [27]. Although some ATL cells express the regulatory T‐cell‐transcription factor FOXP3, FOXP3‐positive and negative cases did not differ significantly in survival [28, 29, 30]. ATL cells may also express Th1‐ and/or Th2‐transcription factors/chemokine receptors, but the prognostic relevance of the IHC‐based classification of TBX21 and GATA3 subtypes in ATL remains unclear.

In this study, we analyzed the prognostic significance of IHC‐based TBX21 and GATA3 subtype classification in a cohort of indolent ATL patients with cutaneous lesions.

## 2 Methods

### 2.1 Study Design, Patients, and Clinical Data Collection

This was a single‐center, retrospective cohort study involving 47 indolent ATL patients with cutaneous lesions, newly diagnosed at the Department of Dermatology, Kagoshima University Hospital, between January 1998 and December 2009. The diagnosis of ATL was based on the positive serum anti‐HTLV‐1 antibodies, detection of clonal integration of HTLV‐1 proviral DNA in the peripheral blood and/or cutaneous lesions, and histopathological confirmation of cutaneous involvement by mature CD4‐positive T‐cell lymphoma.

We reviewed medical charts, clinical photographs, and histopathological slides of the patients and collected the following data at diagnosis: age, sex, clinical subtype, type of cutaneous lesions, and serum sIL‐2R level. Clinical course and outcome of each patient at the end of the follow‐up period in December 2012 were also recorded.

Only patients with smoldering‐type ATL and chronic‐type ATL without unfavorable prognostic factors [3], according to the Shimoyama criteria [2], were included. In this study, PCT type was analyzed separately from other smoldering types with non‐tumoral cutaneous lesions. PCT type was defined as smoldering type with at least one cutaneous tumor and no lung involvement. Cutaneous tumor was defined as a solid lesion ≥ 1 cm in diameter, with evidence of deep infiltration in the skin (vertical growth). In this study, four cases with ≥ 5% abnormal lymphocytes in peripheral blood but without lymphocytosis (≥ 4000/μL) were classified as PCT type. None of the patients had lung lesions.

Cutaneous lesions were classified into six types according to the previous study [19]: patch, plaque, nodulotumoral, erythrodermic, multipapular, and purpuric. In our cohort, however, no patient had purpuric‐type cutaneous lesions.

Based on the iATL‐PI [13], patients were classified into three risk groups: low‐risk, sIL‐2R ≤ 1000 U/mL; intermediate‐risk, 1000 U/mL < sIL‐2R ≤ 6000 U/mL; and high‐risk, 6000 U/mL < sIL‐2R. In the modified iATL‐PI [16], the intermediate‐risk group of the original iATL‐PI was further subdivided into low‐intermediate‐risk group, sIL‐2R < 3000 U/mL; and high‐intermediate‐risk group, 3000 U/mL ≤ sIL‐2R. The sIL‐2R levels at diagnosis were unavailable in six patients.

The study was approved by the ethics committee of Kagoshima University Graduate School of Medical and Dental Sciences (approval number H23‐215) and was conducted in accordance with the Declaration of Helsinki. The requirement for written informed consent was waived due to the retrospective nature of the study. Instead, an opt‐out approach was used, and the opportunity to decline participation was provided through public disclosure.

### 2.2 Treatment

We selected the study period to minimize potential confounding effects of newer therapeutic interventions on the survival of patients, because several novel agents for ATL became available or approved in Japan after 2012 [7, 31]. As a result, only one patient received mogamulizumab after progression to acute type and subsequent relapse. IFN/AZT, lenalidomide, valemetostat, histone deacetylase inhibitors, bexarotene, or brentuximab vedotin were not given to any patients during the study period. All patients were initially treated with skin‐directed therapies. Multiagent combination chemotherapy was not administered before progression to aggressive ATL, except in two patients with progressive PCT‐type ATL.

### 2.3 IHC and Subtype Classification

Formalin‐fixed, paraffin‐embedded tissue sections were deparaffinized and rehydrated. Antigen retrieval was performed by autoclaving at 121°C for 15 min in 0.01 M sodium citrate buffer (pH 6.0). Sections were stained using the Nichirei Histofine Simple Stain MAX‐PO kit (Nichirei, Tokyo, Japan). Diaminobenzidine was used as the substrate, with hematoxylin as a counterstain.

In addition to the standard panel of IHC markers for cutaneous T‐cell lymphomas, we performed IHC using the following antibodies: FOXP3 (236A/E7, eBioscience, Waltham, MA), T‐bet/TBX21 (4B10, Santa Cruz, Dallas, TX), CXCR3 (1C6, BD Biosciences, Franklin Lakes, NJ), GATA3 (HG3‐31, Santa Cruz), and CCR4 (KM2160, Kyowa Kirin, Tokyo, Japan).

According to the previous studies [29, 30], the cutoff value for FOXP3 positivity was set at > 30% of tumor cells. For the classification of TBX21 and GATA3 subtypes, the IHC algorithm for PTCL NOS [27] was applied. If tumor cells were > 20% positive for TBX21 or CXCR3, they were classified as the TBX21 subtype. In cases not classified as TBX21 subtype, if tumor cells were > 50% positive for GATA3 or CCR4, they were classified as the GATA3 subtype. All cases in our cohort were classified into either TBX21 or GATA3 subtype, and no case was categorized as unclassified.

### 2.4 Statistical Analysis

Overall survival (OS) was defined as the time from the initial diagnosis to death from any cause. Patients who survived beyond the end of the follow‐up period were censored at the date of the last contact before December 2012. Progression‐free survival (PFS) was defined as the time from the initial diagnosis to the date of the last contact before progression to aggressive ATL or before death from any cause. Surviving patients without progression were censored at the date of the last contact before December 2012.

Survival curves were estimated using the Kaplan–Meier method [32], and differences between groups were assessed using the log‐rank test [33]. The independent prognostic impact of variables was evaluated by multivariate analyses using the Cox proportional hazards regression model [34].

All reported *p* values are two‐tailed, with values less than 0.05 considered statistically significant. To control the overall significance level at 0.05, the Bonferroni method [35] was applied for pairwise multiple comparisons. Statistical analyses were performed using JMP software (SAS Institute, Cary, NC, USA).

## 3 Results

### 3.1 Characteristics of the Patients and Survival for the Entire Group

A cohort of 47 indolent ATL patients with cutaneous lesions was analyzed in this study. Characteristics of the patients are summarized in Table 1. During the median follow‐up period of 18.1 months (range, 3.8–93.2 months), 22 patients died. Progression to aggressive ATL occurred in 22 patients during the follow‐up period. Median OS was 55.8 months (range, 5.8–91.9 months), and median PFS was 17.4 months (range, 1.8–73.2 months).

**TABLE 1:** Patient characteristics.

Characteristic	Median (range)	*n*
Age, years	71 (31–93)	47
Sex		
Female		17
Male		30
Clinical subtype		
Smoldering		25
Chronic		5
PCT		17
Type of skin lesions		
Patch		4
Plaque		5
Nodulotumoral		21
Erythrodermic		2
Multipapular		15
iATL‐PI		
Low		12
Intermediate		26
High		3
Modified iATL‐PI		
Low‐risk		12
Low‐intermediate‐risk		20
High‐intermediate‐risk		6
High‐risk		3
FOXP3		
Negative		29
Positive		18
IHC subtype		
TBX21		33
GATA3		14

Abbreviations: iATL‐PI, indolent adult T‐cell leukemia‐lymphoma prognostic index; IHC, immunohistochemistry; PCT, primary cutaneous tumoral.

### 3.2 Survival According to the Clinical Subtype and Type of Skin Lesions

**FIGURE 1:**
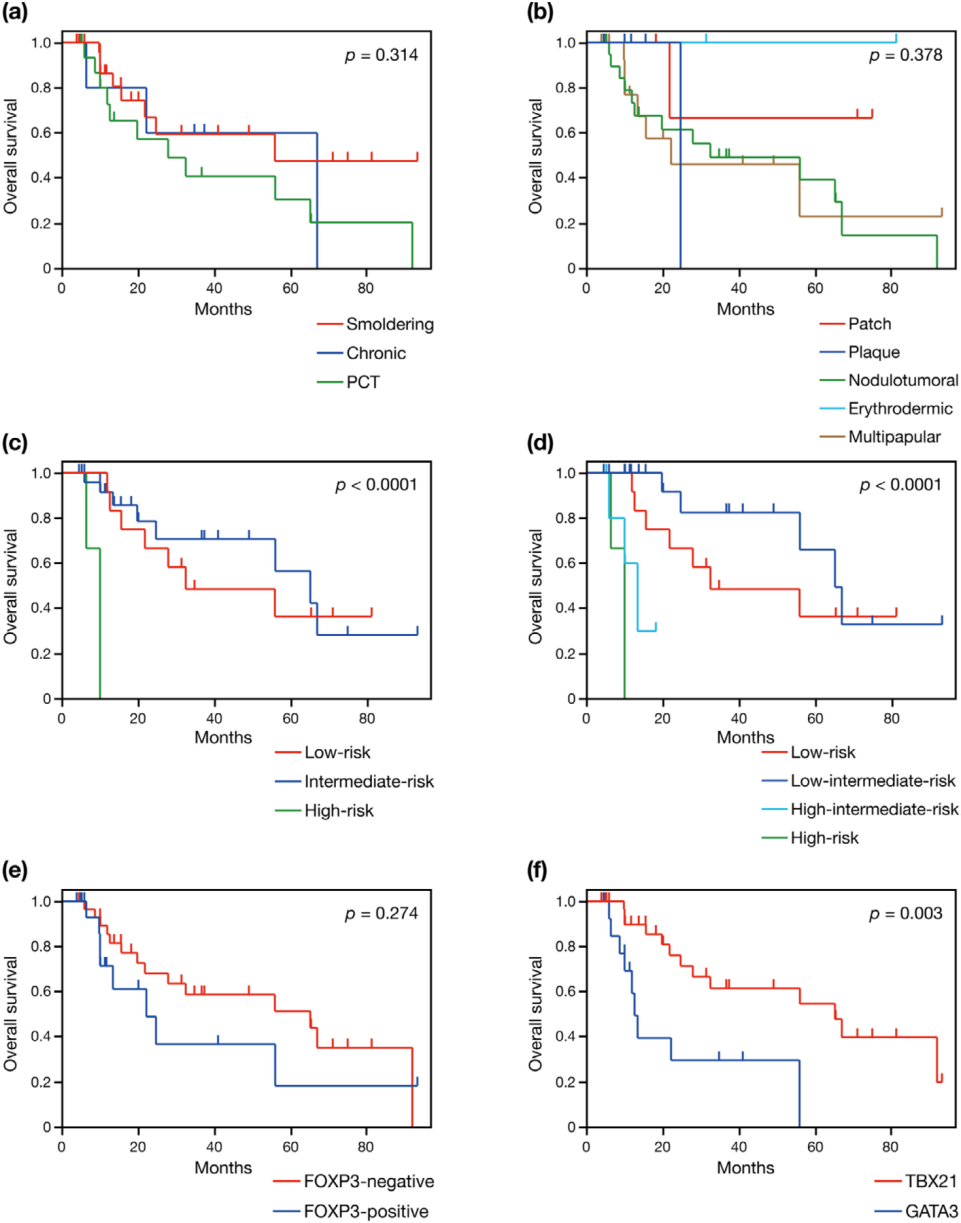
Overall survival according to the clinical subtype (a), type or skin lesions (b), iATL‐PI (c), modified iATL‐PI (d), FOXP3 expression (e), and IHC subtype (f). iATL‐PI, indolent adult T‐cell leukemia‐lymphoma prognostic index; IHC, immunohistochemistry; PCT, primary cutaneous tumoral.

In this study, we subclassified the indolent ATL into smoldering type (excluding PCT type), favorable chronic type, and PCT type, based on previous reports indicating that PCT type is associated with poor prognosis [15, 18, 20, 21]. In our cohort, no statistically significant differences in OS were observed between patients with PCT type and those with smoldering type (*p* = 0.153), chronic type (*p* = 0.620), or both (*p* = 0.153) in univariate analyses; although survival tended to be worse in PCT type (Figure 1a). No significant relationship between the type of cutaneous lesions and survival was observed (Figure 1b).

### 3.3 Survival According to the iATL‐PI and Modified iATL‐PI

According to the iATL‐PI based on the sIL‐2R levels [13], we found significant differences in OS between low‐ and high‐risk groups (*p* < 0.0001), and between intermediate‐ and high‐risk groups (*p* = 0.0004) (Figure 1c). There was no significant difference in OS between low‐ and intermediate‐risk groups (*p* = 0.677) (Figure 1c). When applying the modified iATL‐PI [16], in which the intermediate‐risk group of the original iATL‐PI is subdivided into low‐intermediate‐ and high‐intermediate‐risk groups, the high‐intermediate‐risk group showed significantly worse OS compared to the low‐risk group (*p* = 0.0414) and the low‐intermediate‐risk group (*p* = 0.0001) (Figure 1d). No significant difference was observed between high‐intermediate‐ and high‐risk groups (*p* = 0.202) (Figure 1d). These results suggest that the modified iATL‐PI may be more suitable than the original iATL‐PI for identifying patients with less favorable prognosis.

### 3.4 Survival According to the IHC Subtype

**FIGURE 2:**
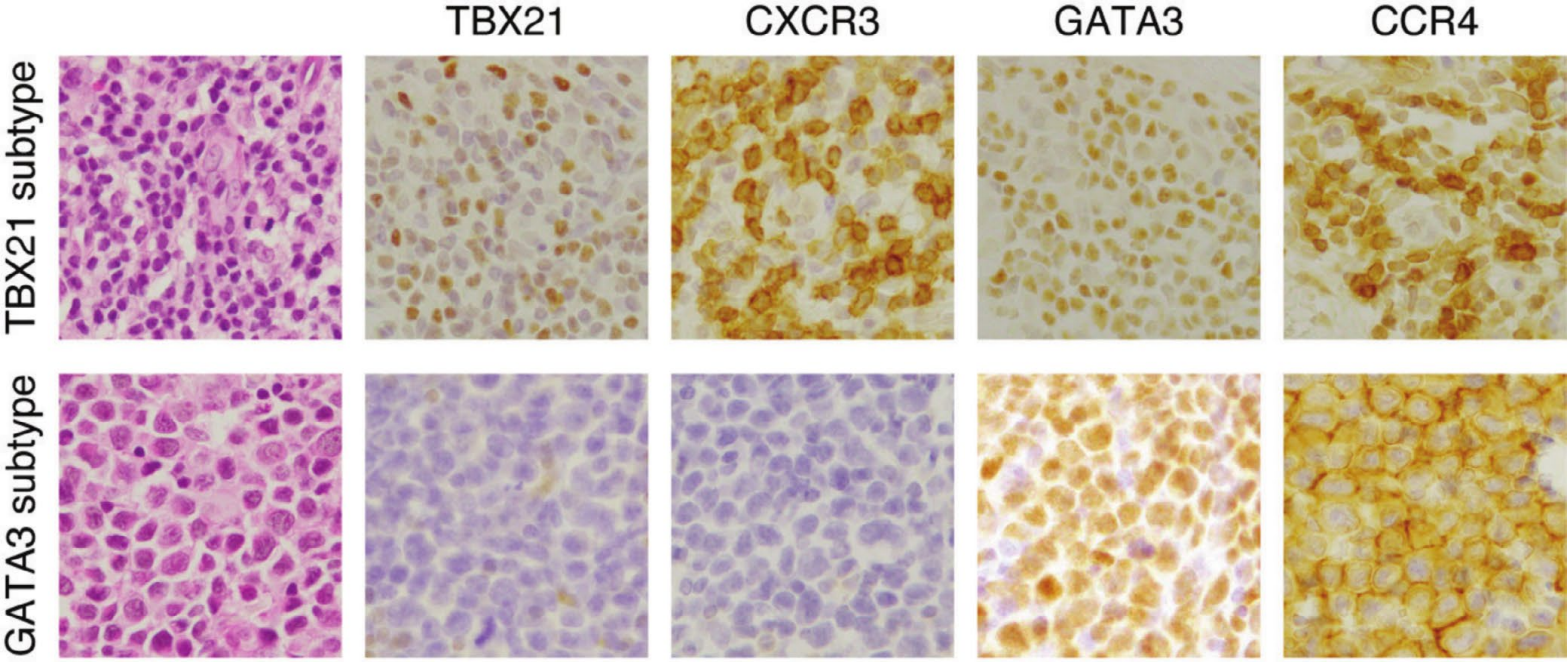
Representative immunohistochemistry staining for TBX21, CXCR3, GATA3, and CCR4. Positive staining for TBX21 (nuclear) and CXCR3 (membranous) in a case of TBX21 subtype (upper panels). This case is also positive for GATA3 (nuclear) and CCR4 (membranous). Negative staining for TBX21 and CXCR3 and positive staining for GATA3 and CCR4 in a case of GATA3 subtype (lower panels). Original magnification 1000×.

Consistent with previous studies [28, 29, 30], we found no statistically significant difference in OS between FOXP3‐positive and negative cases (Figure 1e). In contrast, patients with TBX21 subtype showed significantly better OS compared to those with GATA3 subtype (Figure 1f). Among the 47 cases, 14 and 29 cases were positive for TBX21 and CXCR3 (> 20%), respectively, and 30 and 42 cases were positive for GATA3 and CCR4 (> 50%), respectively. Within the 33 cases classified as TBX21 subtype, 20 and 30 cases were positive for GATA3 and CCR4, respectively (Figure 2).

### 3.5 Prognostic Significance of the TBX21 and GATA3 Subtype Classification

To evaluate the independent prognostic value of the IHC‐based TBX21 and GATA3 subtype classification for predicting survival, we conducted multivariate analyses using known prognostic factors for indolent ATL [15, 16, 18, 20, 21, 36] as covariates. A multivariate analysis incorporating age, clinical subtype, modified iATL‐PI, and IHC subtype demonstrated that all variables, including IHC subtype, were independently significant predictors of OS (Table 2).

**TABLE 2:** Multivariate analysis for overall survival.

Variable	HR	95% CI	*p*
Age			
Continuous	1.056	1.007–1.122	0.042
Clinical subtype			
Smoldering/chronic	1.00		
PCT	4.535	1.150–22.412	0.040
Modified iATL‐PI			
Low‐risk	1.00		
Low‐intermediate‐risk	0.322	0.066–1.268	0.124
High‐intermediate‐risk	18.316	2.562–154.524	0.004
High‐risk	51.129	3.472–1165.779	0.006
IHC subtype			
TBX21	1.00		
GATA3	6.824	1.578–34.859	0.013

Abbreviations: CI, confidence interval; HR, hazard ratio; iATL‐PI, indolent adult T‐cell leukemia‐lymphoma prognostic index; IHC, immunohistochemistry; PCT, primary cutaneous tumoral.

We also performed a multivariate analysis using PFS as the endpoint to identify predictors of progression. When clinical subtype, modified iATL‐PI, and IHC subtype were included as variables, IHC subtype remained an independent predictor of PFS, alongside PCT type and high‐intermediate‐ and high‐risk groups (Table 3).

**TABLE 3:** Multivariate analysis for progression‐free survival.

Variable	HR	95% CI	*p*
Clinical subtype			
Smoldering/chronic	1.00		
PCT	3.146	1.132–9.008	0.028
Modified iATL‐PI			
Low‐risk	1.00		
Low‐intermediate‐risk	0.762	0.270–2.214	0.606
High‐intermediate‐risk	6.739	1.503–28.446	0.009
High‐risk	11.786	1.456–71.245	0.009
IHC subtype			
TBX21	1.00		
GATA3	3.874	1.301–11.264	0.012

Abbreviations: CI, confidence interval; HR, hazard ratio; iATL‐PI, indolent adult T‐cell leukemia‐lymphoma prognostic index; IHC, immunohistochemistry; PCT, primary cutaneous tumoral.

## 4 Discussion

We showed that GATA3 subtype is an independently significant prognostic factor for OS in indolent ATL with cutaneous lesions, along with older age, PCT type, and high‐intermediate‐ and high‐risk groups of the modified iATL‐PI. We also confirmed that GATA3 subtype serves as a predictor of PFS, independent of PCT type and the modified iATL‐PI.

In a previous study [17], CCR4 expression on more than 10% of tumor cells was shown to be associated with cutaneous involvement and to serve as an independent prognostic factor for all clinical subtypes of ATL. In our study population, however, all cases expressed CCR4 on more than 10% of tumor cells. In another study [30], no significant difference in OS was observed between positive (≥ 30% of tumor cells) and negative cases for any of the following markers: TBX21, CXCR3, GATA3, or CCR4, among patients with acute‐ and lymphoma‐type ATL. That study, however, employed different cutoff values and analyzed the prognostic impact of each marker individually, rather than utilizing the subtype classification according to the IHC algorithm. Therefore, it will be important to investigate the prognostic relevance of the IHC‐based classification in other clinical subtypes of ATL, beyond indolent ATL with or without cutaneous lesions.

This study did not explore genetic alterations, although genomic aberrations associated with prognosis in smoldering ATL with cutaneous lesions were reported [23]. PTCL‐TBX21 and PTCL‐GATA3 subtypes were originally identified based on gene expression profiling and are known to be driven by distinct oncogenic pathways [26]. ATL exhibits gene expression signatures distinct from those of both PTCL‐TBX21 and PTCL‐GATA3 subtypes [26, 37]. While less than 50% of PTCL‐TBX21 cases classified using gene expression profiling were positive for GATA3 (42%) and/or CCR4 (45%) by IHC [27], the majority of the TBX21 subtype in our cohort were positive for GATA3 (61%) and/or CCR4 (91%). It remains unclear whether IHC‐based TBX21 and GATA3 subtypes in ATL are associated with specific gene alterations and/or distinct gene expression profiles.

For the IHC‐based classification of PTCL NOS, cases with a T follicular helper (TFH) phenotype should be excluded [27, 37, 38]. In this study, we did not assess the expression of TFH markers in most cases. However, recent studies indicate that more than 10% of ATL cases exhibit a TFH phenotype [30, 39]. Although the prognosis of ATL cases with a TFH phenotype was not significantly different from that of other ATL cases [39], it is possible that the TFH subtype of ATL represents a distinct subgroup from TBX21 and GATA3 subtypes and should be considered separately in future studies. Interestingly, the GATA3 subtype was also associated with poor prognosis in patients with nodal TFH cell lymphoma other than angioimmunoblastic type [40].

In addition to the issues discussed above, this study has several limitations. First, the sample size was relatively small due to the rarity of the disease and the retrospective nature of data collection from a single institution. Second, several novel agents have recently been used for the treatment of ATL, which may influence survival and/or progression [7, 31]. Third, in cutaneous lesions of ATL, it sometimes is not easy to distinguish tumor cells from reactive cells, although the IHC data in this study were validated through independent evaluation by two investigators (K.K. and Y.U.).

In conclusion, our findings demonstrate that IHC‐based TBX21 and GATA3 subtype classification is useful for predicting survival and progression in indolent ATL patients with cutaneous lesions. The prognostic significance of these IHC subtypes should be validated in larger, prospective cohort studies.

## Ethics Statement

The study was approved by the ethics committee of Kagoshima University Graduate School of Medical and Dental Sciences (approval number H23‐215).

## Consent

The requirement for written informed consent was waived due to the retrospective nature of the study. Instead, an opt‐out approach was used, and the opportunity to decline participation was provided through public disclosure.

## Conflicts of Interest

Kazuhiro Kawai is an Editorial Board member of The Journal of Dermatology and a co‐author of this article. To minimize bias, he was excluded from all editorial decision‐making related to the acceptance of this article for publication. Other authors declare no conflicts of interest for this article.

## Data Availability

The data that support the findings of this study are available from the corresponding author upon reasonable request.
